# 5 years of psychiatric high intensive care in Belgium: lessons learned

**DOI:** 10.1192/j.eurpsy.2026.10391

**Published:** 2026-07-31

**Authors:** R. Bruffaerts, Katrien Vandenhout, Leontien Jansen, Camille Kubicek, Savanah Héréus, Carmela Scibetta, Hella Demunter

**Affiliations:** Universitair Psychiatrisch Centrum - Ku Leuven, Leuven, Belgium

## Abstract

**Abstract:**

Amid ongoing mental healthcare reforms in Belgium, federal authorities initiated a significant transformation of inpatient care for individuals with severe mental illness from 2020 onwards, leading to the transition of 31 psychiatric units across Belgium into high-intensive care (HIC) units (Bruffaerts et al., 2025). In this presentation we discuss (1) clinical and sociodemographic profiles of patients admitted to HIC units in Belgium; (2) findings on the role of HIC units in the provision of appropriate care for patients in severe crisis (including met and unmet needs for treatment and the extent to which HIC patients have been treated in a crisis care pathway); (3) pre-/post comparisons in patients’ outcomes. Using a value-based framework, we provide preliminary evidence on the role HIC units may play in the development of a delivery system for people in crisis.

**Disclosure of Interest:**

None Declared

